# Protection against tuberculosis achieved by dissolving microneedle patches loaded with live *Mycobacterium paragordonae* in a BCG prime-boost strategy

**DOI:** 10.3389/fimmu.2023.1178688

**Published:** 2023-06-16

**Authors:** Mi-Hyun Lee, Hyejun Seo, Moon-Su Lee, Byoung Jun Kim, Hye Lin Kim, Du Hyung Lee, Jaehun Oh, Ju Yeop Shin, Ju Young Jin, Do Hyeon Jeong, Bum-Joon Kim

**Affiliations:** ^1^ Department of Microbiology and Immunology, College of Medicine, Seoul National University, Seoul, Republic of Korea; ^2^ Department of Biomedical Sciences, College of Medicine, Seoul National University, Seoul, Republic of Korea; ^3^ BK21 FOUR Biomedical Science Project, Seoul National University College of Medicine, Seoul, Republic of Korea; ^4^ Cancer Research Institute, College of Medicine, Seoul National University, Seoul, Republic of Korea; ^5^ Institute of Endemic Diseases, Seoul National University Medical Research Center (SNUMRC), Seoul, Republic of Korea; ^6^ Medical Business Division, Raphas Co., Ltd., Seoul, Republic of Korea; ^7^ Liver Research Institute, College of Medicine, Seoul National University, Seoul, Republic of Korea

**Keywords:** *Mycobacterium tuberculosis*, *Mycobacterium paragordonae*, microneedle patch, BCG booster vaccine, droplet extension method

## Abstract

**Introduction:**

Skin vaccination using dissolving microneedle patch (MNP) technology for transdermal delivery is a promising vaccine delivery strategy to overcome the limitations of the existing vaccine administration strategies using syringes. To improve the traditional microneedle mold fabrication technique, we introduced droplet extension (DEN) to reduce drug loss. Tuberculosis remains a major public health problem worldwide, and BCG revaccination had failed to increase the protective efficacy against tuberculosis. We developed an MNP with live *Mycobacterium paragordonae* (Mpg) (Mpg-MNP) as a candidate of tuberculosis booster vaccine in a heterologous prime-boost strategy to increase the BCG vaccine efficacy.

**Materials and methods:**

The MNPs were fabricated by the DEN method on a polyvinyl alcohol mask film and hydrocolloid-adhesive sheet with microneedles composed of a mixture of mycobacteria and hyaluronic acid. We assessed the transdermal delivery efficiency by comparing the activation of the dermal immune system with that of subcutaneous injection. A BCG prime Mpg-MNP boost regimen was administered to a mouse model to evaluate the protective efficacy against *M. tuberculosis*.

**Results:**

We demonstrated the successful transdermal delivery achieved by Mpg-MNP compared with that observed with BCG-MNP or subcutaneous vaccination *via* an increased abundance of MHCII-expressing Langerin+ cells within the dermis that could migrate into draining lymph nodes to induce T-cell activation. In a BCG prime-boost regimen, Mpg-MNP was more protective than BCG-only immunization or BCG-MNP boost, resulting in a lower bacterial burden in the lungs of mice infected with virulent *M. tuberculosis*. Mpg-MNP-boosted mice showed higher serum levels of IgG than BCG-MNP-boosted mice. Furthermore, Ag85B-specific T-cells were activated after BCG priming and Mpg-MNP boost, indicating increased production of Th1-related cytokines in response to *M. tuberculosis* challenge, which is correlated with enhanced protective efficacy.

**Discussion:**

The MNP fabricated by the DEN method maintained the viability of Mpg and achieved effective release in the dermis. Our data demonstrate a potential application of Mpg-MNP as a booster vaccine to enhance the efficacy of BCG vaccination against *M. tuberculosis*. This study produced the first MNP loaded with nontuberculous mycobacteria (NTM) to be used as a heterologous booster vaccine with verified protective efficacy against *M. tuberculosis.*

## Introduction

Tuberculosis has been a persistent global challenge since it emerged as an epidemic in Europe in the 18^th^ century (1). Indeed, since the introduction of the Bacillus Calmette-Guérin (BCG) vaccine based on an attenuated BCG strain in 1921, a BCG vaccination campaign has been implemented around the world, and the actual tuberculosis incidence rate is decreasing (2). However, 10 million people fell ill with TB and 1.5 million people died due to TB in 2020; TB is still ranked as one of the top 10 causes of death and is considered the deadliest infectious disease worldwide (3, 4). Although BCG is the only licensed vaccine for TB and has 70-80% efficacy against childhood TB, it has shown limited effectiveness against adult TB (5).

Since worldwide BCG vaccine coverage reached over 90% in 2018, a prime-boost vaccination strategy has been considered an efficient vaccine-enhancing strategy to enhance the effectiveness of BCG (6, 7). However, there is a lack of evidence concerning the efficacy of BCG revaccination *via* repeated BCG doses in protecting against TB (8). To overcome the limitations of BCG vaccination, exposure to nontuberculous mycobacteria (NTM) could be a solution to enhance the protective effect of BCG vaccination. A recent study demonstrated that exposure to *M. avium*, an environmental NTM strain, increases T-cell activation in branchial lymph nodes and improves the protective effects of BCG vaccination by inducing Th1 and Th17 immune responses (9, 10). In addition, the SRL172 strain of *M. vaccae*, an inactivated environmental NTM, is the only new tuberculosis booster vaccine candidate considered to show efficacy in a phase 3 trial (11). These findings highlight the potential of developing a BCG booster vaccine using NTM strains.

Previously, we reported that a temperature-sensitive NTM, *Mycobacterium paragordonae* (Mpg), is a potent tuberculosis vaccine candidate. Mpg induced enhanced protective immune responses against *M. tuberculosis* and *M. abscessus* infection by eliciting a Th1 immune response (12). Moreover, the development of recombinant Mpg expressing human immunodeficiency virus (HIV)-1 p24 or the severe acute respiratory syndrome coronavirus 2 (SARS-CoV-2) RBD has shown the potential of Mpg as a delivery vector for vaccines (13, 14). Based on the high immunogenicity of Mpg, we suggest Mpg as a potent booster vaccine after BCG priming against tuberculosis.

A transdermal delivery strategy can be effective in managing infectious diseases (15). The epidermis and dermis contain professional antigen-presenting cells (APCs) that can initiate adaptive immunity effectively by migrating to draining lymph nodes (16). The microneedle drug delivery system has been considered a promising platform to complement hypodermic injection (17). In particular, hyaluronic acid (HA), which has been approved by the US FDA, has been widely used to dissolve microneedle components due to its biodegradability, biocompatibility, and skin permeability (18, 19). In this study, Mpg-MNP was manufactured with HA using Raphas’ proprietary droplet extension method (DEN) (20, 21). The DEN method provides a gentle manufacturing environment without heat or UV irradiation because blowing air is applied directly to the polymer droplet to solidify a microneedle shape.

In this study, we developed a safe and painless BCG booster vaccine by fabricating Mpg-MNP and demonstrated the enhanced protective efficacy of Mpg-MNP in a virulent *M. tuberculosis* infection model. We also evaluated the protective effects of strengthened cellular immune responses, including induction of Th1-biased immunity *via* successful transdermal delivery of live Mpg.

## Materials and methods

### Mycobacterial strains and culture

The mycobacterial strains used were as follows: *Mycobacterium paragordonae* (Mpg) JCM 18565^T^, *Mycobacterium bovis* BCG Tokyo strain, *Mycobacterium tuberculosis* strain H37Ra ATCC 25177, and *M. tuberculosis* K-strain NCCP 15986. The pathogen resources (NCCP 15986) for this study were provided by the National Culture Collection for Pathogens. The strains were cultured from frozen stocks (−80°C) and subcultured in 7H9 broth with 2.5% glycerol, 0.2% Tween-80, and 10% ADC or on 7H10 agar plates with 0.5% glycerol and 10% OADC at 37°C or 30°C (in the case of Mpg).

### Fabrication of Mpg*-*loaded MNPs *via* the DEN method

Mpg cultured in 7H9-ADC was provided by the Institute of Medicine of Seoul University (Seoul, Korea) and was harvested and washed two times with phosphate-buffered saline (PBS) (pH 7.4). After washing, Mpg was reconstructed in PBS and mixed with sodium hyaluronate. (HA, EP-1, Bloomage Freda Biopharm Co Ltd, China) (25%, wt). This mixture was homogenized with a planetary centrifugal mixer (ARV-310, THINKY Corp., Japan) at room temperature. The temperature during MNP production can rise up to 45 to 50˚C which does not affect the activation of used mycobacterial strains. The DEN method was used to prepare Mpg-loaded MNPs. The mixture was dropped onto polyvinyl alcohol (PVA) mask film and hydrocolloid-based adhesive, which was dispensed onto an adhesive patch by a solution dispenser (Super Sigma CMII, Musashi, Japan). Then, solid-state microneedles were fabricated from the liquid-phase mixture through elongation and air-blowing processes. Each patch was designed as 24 microneedle arrays (needle pitch, 1.4 mm; needle length, 0.6 mm).

### Physical strength of Mpg-loaded MNPs

The mechanical fracture force was measured with a universal testing machine (Z0.5TN, Zwick/Roell, Germany). After a single microneedle was attached to the rigid stainless-steel station, microneedles were pressed by the sensor probe at a speed of 1.1 mm/s for axial force application. The axial force required to move the probe was tracked by the universal testing machine as a function of distance. The maximum force before the force drop was measured as the axial fracture force for Mpg-MNPs 0.6mm in length.

### Analysis of mycobacterial encapsulation and viability in MNPs

A single MNP was immersed in 1 ml of PBS at room temperature for two hours to elute mycobacteria loaded on the patch. The eluted samples were centrifuged, and the pellets were used to confirm the number and viability of MNP-loaded mycobacteria through real-time quantitative PCR (RT−qPCR) and ATP bioluminescence assays.

To quantify the number of MNP-loaded mycobacteria through RT−qPCR, 0.1, 0.5, 1, 5, and 10 x 10^6^ CFU of mycobacteria (standard) and the eluted pellets from MNPs were disrupted by a mini-bead beater with TRIzol™ (Invitrogen, Massachusetts, USA) and glass beads (0.1~0.3 mm). Then, the lysates were centrifuged into three aqueous layers, and DNA was extracted *via* the manufacture’s protocol for TRIzol™. Briefly, the DNA samples were isolated from the intermediate layer using an ethanol precipitation method and used as templates. RT−qPCR was performed with a SensiFAST™ SYBR Lo-ROX One-Step kit (Bioline, London, UK) and *hsp65* primers (Forward primer: 5’- GTCGAGGAGTCCAACACCTT- 3’; Reverse primer: 5’-GAGCTGACCAGCAGGATGTA-3’) to quantify the number of MNP-loaded mycobacteria. The standardization of RT−qPCR was carried out with DNA from mycobacterial samples (standard). The number of the MNP-loaded bacteria was determined based on a standard curve of Cq value of the *hsp65* versus the log CFU (**Supplementary Figure 1A**). The eluted pellets of MNPs were reacted with a 3M™ Clean-Trace™ Water Plus – Total ATP kit (3 M, Minnesota, USA) to check the viability of MNP-loaded mycobacteria. The reaction mixture was transferred to a Nunclon™ 96-well white flat-bottom plate (Invitrogen, Massachusetts, USA) to measure the luminescence, which reveals the amount of ATP in the sample, indicating viability.

### *In vivo* experimental models

Seven-week-old female BALB/c mice were purchased from Orientbio (Seoul, South Korea) and used for experiments at the age of 8 weeks. In the prime-boost vaccination protocol, all mice were anesthetized prior to subcutaneous injection of BCG (1×10^6^ CFUs in 100 μl of PBS). The hair on the back was completely removed with an electric shaver and hair removal cream. MNPs were applied on the back and wrapped around the torsos of the mice, and MNPs were removed after 24 hr. In a virulent *M. tuberculosis* (MTB) K-strain challenge model, MTB (1×10^6^ CFUs in 100 μl of PBS) was injected through the tail vein of mice after anesthetization. All mice were sacrificed by CO_2_ asphyxiation.

### Isolation of primary cells

After isolation, the skin-draining lymph nodes, which were axillary, lateral axillary, and inguinal lymph nodes, were incubated in 750 µL of a 1 mg/mL collagenase IV (Sigma−Aldrich, Massachusetts, USA) and 0.2 mg/mL DNase I (Sigma−Aldrich, Massachusetts, USA) mixture at 37°C for 30 minutes with shaking to digest connective tissue, and the reaction was terminated by adding 100X EDTA (7.5 µL). Spleens and digested lymph nodes were homogenized using a 70 µm cell strainer (SPL, Pocheon, Republic of Korea) and the plunger from a 5 cc syringe and centrifuged. To prevent contamination of red blood cells, the pellets of each organ were treated with red blood cell lysis buffer (Sigma−Aldrich, Massachusetts, USA), and the reaction was terminated by adding complete RPMI [supplemented with 10% fetal bovine serum (FBS)] twice. After centrifugation, the isolated primary cells were used for further experiments.

### Cytokine assay

The isolated primary cells of lymph nodes or spleens were cultured (1x10^6^ cells/well) in 96-well round-bottom plates. Then, the cells were restimulated with 5 µg/mL of H37Ra lysate, which was a sonicated sample of *M. tuberculosis* strain H37Ra, or 5 µg/mL of the Ag85B protein, which was produced by *Escherichia coli*, for 3 days, and IL-10, IL-12p40, TNF-α, and IFN-γ levels in the supernatant were measured using an Invitrogen cytokine ELISA kit. For IFN-γ ELISPOT, PVDF membrane-based ELISPOT plates were activated with 75% ethanol and coated with 3 µg/mL anti-mouse IFN-γ capture antibody (100 µL per well; clone: AN-18; Invitrogen, Massachusetts, USA) at 4°C overnight. Then, 1x10^6^ splenocytes per well were loaded onto an ELISPOT plate, which was washed with PBS-T and PBS, and restimulated with 5 µg/mL of H37Ra lysate or Ag85B peptide pool (144-152, IYAGSLSAL; 151-165, ALLDPSQGMGPSLIG; and 262-279, HSWEYWGAQLNAMKGDLQ; each peptide was added at a concentration of 2 µg/mL), which was synthesized by Peptron (Daejeon, Republic of Korea), at 37°C for 20 h. After the washed plate was incubated with anti-mouse-IFN-γ-biotin (3 µg/mL; 100 µL per well; clone: XMG 1.2; Invitrogen, Massachusetts, USA) and then with streptavidin-HRP (BD, New Jersey, USA), spots were developed using an AEC substrate kit (BD, New Jersey, USA). IFN-γ ELISPOT has been described in more detail previously (14).

### Flow cytometry

Primary cells from lymph nodes or spleens were isolated and cultured (1x10^6^ cells/well) in 96-well round-bottom plates. Cells were blocked with anti-CD16/32 (BioLegend, #101301) in buffer (PBS containing 10% FBS and 10 mM EDTA) for Fc receptor blocking for 10 min at RT and stained with surface marker-specific antibodies (1:500) for 30 min on ice. For intracellular cytokine staining, the cells were fixed and permeabilized with cytofix/cytoperm solution (BD biosciences, #BDB554714) for 20 min. After permeabilization, intracellular cytokine-specific antibodies (1:200) were added to the permeabilization buffer and incubated for 40 min on ice. Flow cytometry was performed using BD LSRFortess™ X-20 and the full gating strategy is presented in (**Supplementary Figure 2**). The data were analyzed by FlowJo 10 software and FMO controls were used for gating.

### Histological staining and confocal imaging

The collected skin was stored in 4% paraformaldehyde (PFA) and frozen with optimal cutting temperature compound for cryosectioning. The cryosections were fixed with 4% PFA for 20 min at room temperature and stained with Alexa Fluor^®^ 488 MHCII (Biolegend, #107615), langerin Alexa Fluor^®^ 594 (Santa Cruz, #SC-271272 AF594) or Alexa Fluor^®^ 488 Ki-67 (Biolegend, #151204) antibodies (1:100) overnight at 4°C. The cryosections were mounted with mounting medium containing DAPI (Vectashield, #H-1200), and images were obtained by confocal microscopy (Olympus, FV3000).

For hematoxylin and eosin (H&E) staining, 4% PFA-fixed the lung was embedded in paraffin, and sectioned and made to 5 µm thickness slide. The cryosectioned skin and sectioned lung were stained with hematoxylin and eosin (H&E). The scanned images were analyzed through Aperio ImageScope (CA, USA).

### CFU enumeration assay

The organ lysate was serially diluted in PBS, and the optimally diluted sample was dropped onto a 7H10 agar plate (supplemented with glycerol and OADC). After confirming that the droplet had been dried, the plates were incubated at 37°C with 5% CO_2_ for 3 or 4 weeks to wait for colony formation. The number of CFUs per organ was calculated by considering the number of colonies and the dilution factor.

### Statistical analysis

All experimental data were analyzed by using GraphPad Prism 5 (GraphPad, CA, USA), and every experiment was performed as two or more independent replicates. All results were analyzed by one- or two-way ANOVA with Tukey’s multiple comparison tests to compare multiple experimental groups, and Student’s t-test was applied to compare two experimental groups. The data represent the mean ± standard deviation (SD), and statistical significance is indicated with asterisks as follows: *P < 0.05, **P < 0.01, and ***P < 0.001 (ns, not significant).

## Results

### Fabrication of the dissolved Mpg-MNP and viability of the loaded Mpg

**Figure 1:**
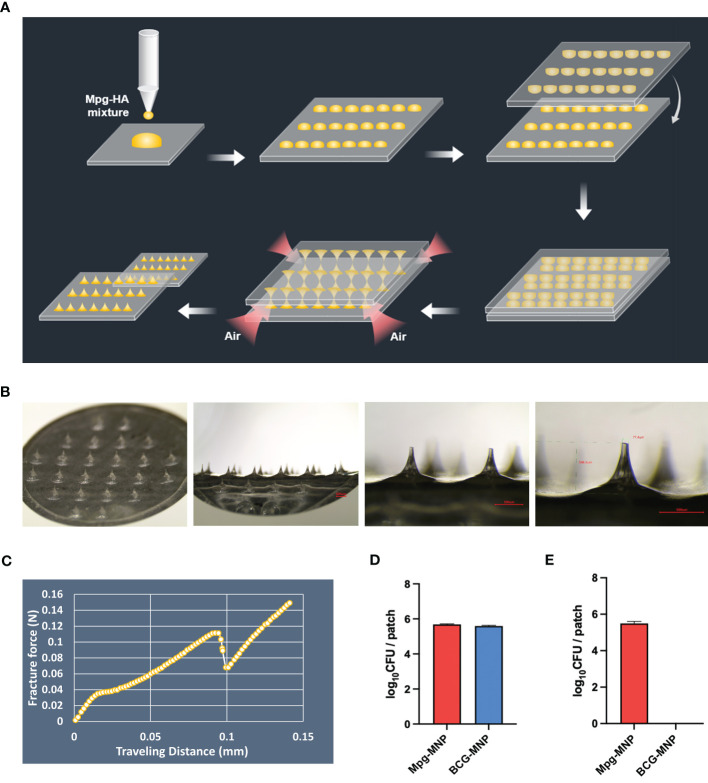
Description of *mycobacterium*-loaded MNPs. **(A)** Schematic illustration of the MNP manufacturing process *via* the DEN method. **(B)** Microscopic images of the microneedle patch at 13.4X, 40X, and 60X magnifications (bar: 500 μm). **(C)** The graph shows the mechanical fracture force of the microneedle patch fabricated by the DEN method. **(D)** The amount of loaded Mpg or BCG on the MNPs was assessed *via* qPCR, and **(E)** the viable Mpg or BCG on the MNPs was measured *via* a CFU assay.

*Mycobacterium*-loaded MNPs were constructed with a mixture of mycobacteria and dissolvable HA *via* the DEN method (**Figure 1A**). The circular hydrocolloid adhesive patches measured 10x10 mm and contained 24 circular cone-shaped microneedles up to ~600 μm in height and ~78μm in tip diameter that could penetrate into the mouse dermis (**Figure 1B**). To examine the transdermal penetration capacity, the mechanical fracture force of a single Mpg-loaded microneedle was measured with a universal testing machine. The axial fracture force was 0.111 N, two times the margin of the minimum average insertion force (0.058 N) of microneedles of this geometry (**Figure 1C** and **Table S1**). We intentionally designed the HA-based Mpg-MNP to quickly achieve transdermal delivery with self-administration.

In addition, we determined the amount of Mpg or BCG on the MNPs *via* a dissolution test using real-time PCR with *hsp65*-specific nested PCR primers. The amount of DNA, which is based on the standard curve between the number of mycobacteria and its Cq value (R^2^ > 0.99, **Supplementary Figure 1A**), extracted from a dissolved Mpg-MNP in PBS showed a 5.68 ± 0.03 log_10_ CFU bacterial load, and that extracted from a BCG-MNP showed a 5.59 ± 0.04 log_10_ CFU bacterial load per patch (**Figure 1D**). However, the opposite trend was observed for the viability of Mpg and BCG on the MNPs. Recovery of live mycobacteria in Mpg or BCG-MNPs was evaluated by confirming CFU and ATP levels after 90 days of storage at 4°C. The CFU log value of the Mpg-MNP was 5.49 ± 0.23 log_10_ CFU, which implies approximately 96.6% viability of Mpg after loading. However, in the case of the BCG-MNP, we did not detect any viable cells after dissolution (**Figure 1E**). Furthermore, we evaluated intracellular ATP in the dissolved solution, and the results of the ATP assay also demonstrated very low levels of bioluminescence in the dissolved BCG-MNP solution, suggesting its poor preservation ability (**Supplementary Figure 1B**). Since we fabricated *mycobacterium*-loaded MNPs without any supplements intended to preserve the viability of the mycobacteria, loss of BCG viability was observed (22). In contrast to BCG, the environmental NTM can survive during starvation and can persist during low-nutrient stress, suggesting that Mpg maintains its high viability after the fabrication of MNPs (23, 24). These results suggest that compared with BCG, Mpg is a suitable strain for HA-based MNP manufacturing *via* the DEN method and for long-term storage.

### Transdermal delivery of Mpg-MNP and activation of the dermal immune system

**Figure 2:**
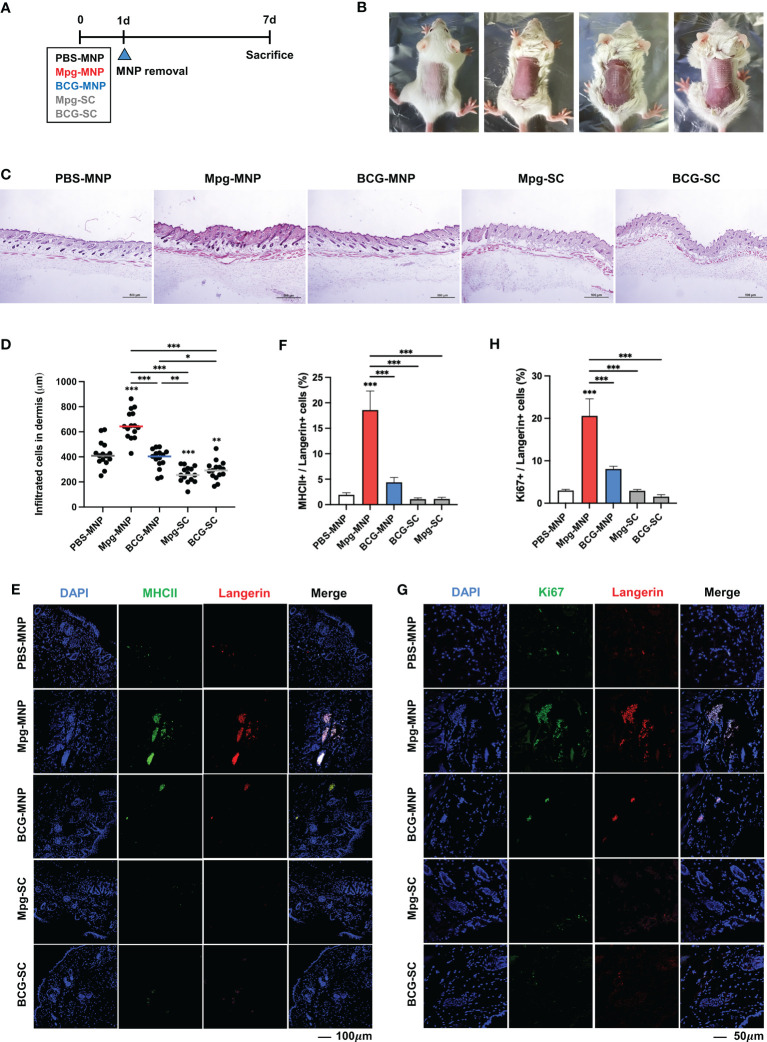
Altered frequencies of immune cells in the dermis after Mpg-MNP application. **(A)** Schematic of the mouse (BALB/c, female, n=5 per group) immunization schedule used to compare the immune responses after MNP application and S.C. injection. **(B)** The process of attaching the microneedle patch to the back of the mouse. **(C)** Representative images of H&E-stained skin after fixing the dorsal skin of mice. **(D)** The counts of infiltrated cells in the dermis are shown as a graph. **(E, G)** Representative confocal microscopy images of mouse dorsal skin. **(E)** Tissue sections were stained for MHCII (green) and Langerin (red) or **(G)** Ki67 (green) and Langerin (red). **(F)** The bar graph shows the frequency of MHC+/Langerin+ cells, which was obtained from **(E)**, and **(H)** the frequency of Ki67+/Langerin+ cells is also shown as a bar graph from **(G)**. Statistical analysis: One-way ANOVA with Tukey’s multiple comparison test, ^*^P < 0.05, ^**^P < 0.01 and ^***^P < 0.001.

The transdermal delivery efficacy of Mpg-MNP was compared with that of BCG-MNP or subcutaneous vaccination *via* a hypodermic syringe (**Figure 2A**). MNPs were applied for 24 h on the backs of female BALB/c mice after removing the hair (**Figure 2B**). Seven days post-vaccination, we harvested the back skin tissues to evaluate alterations in the frequency of immune cells. We found that the Mpg-MNP-treated group showed an increased number of infiltrating cells in the dermis compared to that of all other groups (**Figures 2C, D**). Interestingly, we detected a large subset of activated antigen-presenting cells in the dermis of the Mpg-MNP group; MHCII+ langerin+ cells represented 18.5% of cells in the dermis of the Mpg-MNP-immunized group but only 4.3% of cells in the BCG-MNP group, 1.9% of cells in the PBS-MNP group, 1.1% of cells in the Mpg-SC group and 1.07% of cells in the BCG-SC-immunized group (**Figures 2E, F**). In particular, high levels of Ki-67 expression were found in langerin+ subsets in the dermis, with 20.6% Ki-67+ langerin+ cells after Mpg-MNP application, whereas at most, 8.05% Ki-67+ langerin+ cells were observed in the BCG-MNP group, 2.99% Ki-67+ langerin+ cells were observed in the PBS-MNP group, 2.92% Ki-67+ langerin+ cells were observed in the Mpg-SC group and 1.54% Ki-67+ langerin+ cells were observed in the BCG-SC immunized group (**Figures 2G, H**). Dermal MHCII+ langerin+ cells exhibit migratory capacity, and they proliferate before and after migration (25). Migratory MHCII+ langerin+ cells play an initiating role in the induction of T- or B-lymphocyte-mediated immune responses in the draining lymph nodes (26). Indeed, we found that dermal langerin+ cells in the Mpg-MNP group formed large clusters during proliferation due to the adjacent proliferative Ki-67+ langerin+ cells, which promoted migration (**Figure 2G**) (27, 28). Overall, compared with BCG-MNP or subcutaneous immunization, Mpg-MNP immunization strongly induces migratory MHCII+ langerin+ cells in the dermis that can skew the adaptive immune response.

### Mpg-MNPs induce cellular immune responses in the skin-draining lymph nodes

**Figure 3:**
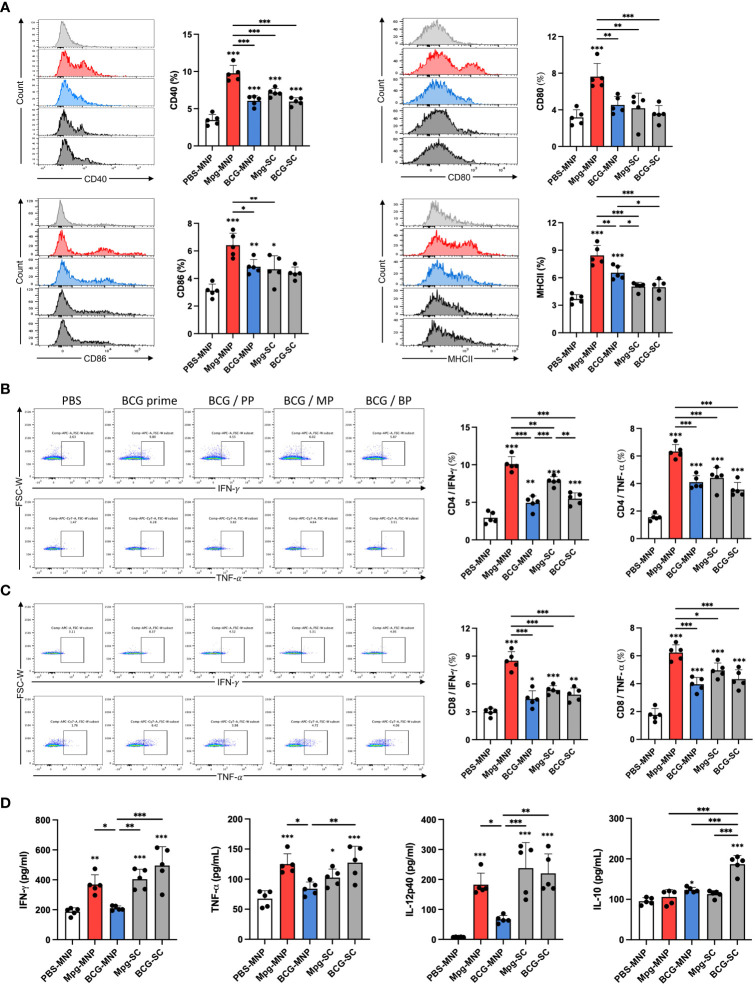
Induction of immune responses in the skin-draining lymph nodes by Mpg-MNP. **(A)** The costimulatory factors CD40^+^ (upper left), CD80^+^ (upper right), CD86^+^ (lower left), and MHCII^+^ (lower right) in CD11b^+^/CD11c^+^ dendritic cells in the draining lymph nodes were analyzed by flow cytometry. **(B, C)** Representative flow cytometry plots and bar graphs of **(B)** CD4+ or **(C)** CD8+ cells releasing IFN-γ (upper panel) and TNF-α (lower panel) in the draining lymph nodes after restimulation with the Ag85B protein for 72 h. **(D)** Cytokine expression levels in the supernatant from lymph nodes restimulated with the Ag85B protein for 72 h were measured by ELISA. The results show the levels of IFN-γ, TNF-α, IL-12p40, and IL-10. The asterisk above the bar indicates statistical significance compared with PBS-MNPs, and the asterisk with a line indicates statistical significance compared with the indicated group. Statistical analysis: One-way ANOVA with Tukey’s multiple comparison test, ^*^P < 0.05, ^**^P < 0.01 and ^***^P < 0.001.

As live Mpg was successfully delivered into the dermis by MNPs, we further analyzed cellular immune responses in the skin-draining lymph nodes. To investigate the induction of adaptive immune responses by migratory antigen-presenting cells from the dermis, we compared the surface profiles of DCs in the draining lymph nodes. Higher expression of CD40, CD80 and CD86 in DCs was observed in response to Mpg-MNP immunization than in response to BCG-MNP, Mpg-SC or BCG-SC immunization, implying the potential to activate T-cell-mediated immune responses (**Figure 3A**). Additionally, upregulated MHCII expression levels in DCs were also identified, suggesting an enhanced ability to present antigens to naïve T cells in response to Mpg-MNP immunization. These data demonstrate the superiority of Mpg-MNP in promoting the maturation of DCs, which is related to the activation of antigen-presenting cells in the dermis.

Next, we assessed CD4+ and CD8+ T-cell priming by mature DCs after restimulation with Ag85B protein for 3 days. FACS analysis of the activation markers, IFN-γ and TNF-α showed that Mpg-MNP immunization increased the frequencies of both active CD4+ and CD8+ T cells in the draining lymph nodes compared with those in the BCG-MNP-immunized group (**Figures 3B, C**). In particular, consistent with our previous study, enhanced antigen-specific IFN-γ secretion potentiated the induction of a Th1-skewed immune response by Mpg-MNP immunization (12). In addition, we performed ELISA to analyze the cytokine profiles on day 3 after restimulation with the Ag85B protein to identify the cytokines secreted into the supernatant. Significantly higher levels of IFN-γ, TNF-α and IL-12p40 were detected in the Mpg-MNP-immunized group than in the BCG-MNP-immunized group, and these levels were correlated with the level of Th1 cell activation. In addition, no noticeable alteration in IL-10 secretion levels was observed in response to Mpg-MNP immunization (**Figure 3D**) (12). Collectively, these data showed that the Mpg-MNP induces both CD4+ and CD8+ T-cell activation by highly mature DCs in the draining lymph nodes, leading to enhanced secretion of Th1 cytokines compared with that observed after BCG-MNP immunization.

### The BCG-prime/Mpg-MNP boost regimen improves protective efficacy against *M. tuberculosis*

**Figure 4:**
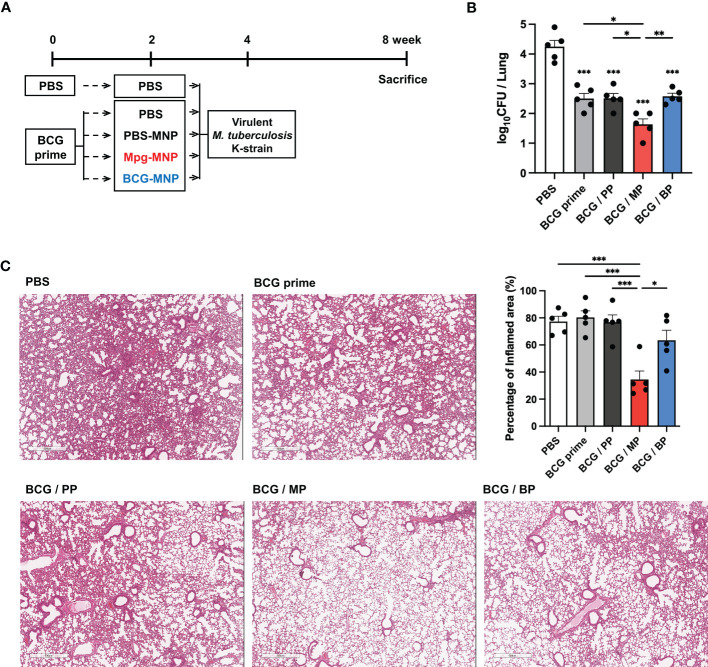
Protective efficacy of a BCG prime/Mpg-MNP boost protocol against virulent *M. tuberculosis in vivo.* **(A)** Schedule used to demonstrate the efficacy of the BCG prime/Mpg-MNP boost strategy against virulent *M. tuberculosis* K-strain challenge (BALB/c, female, n=5 per group). The PBS group was treated with PBS 2 times with a 2-week interval without BCG prime vaccination **(B)** The number of CFUs in the lungs. A representative image of H&E-stained lung lesions is presented in **(C)**. **(C)** The percentage of inflamed area was measured *via* lung histology (BCG prime; BCG prime only, BCG/PP; BCG prime/PBS-MNP boost, BCG/MP; BCG prime/Mpg-MNP boost, BCG/BP; BCG prime/BCG-MNP boost). Statistical analysis: One-way ANOVA with Tukey’s multiple comparison test, ^*^P < 0.05, ^**^P < 0.01 and ^***^P < 0.001.

To verify the protective efficacy of Mpg-MNP against *M. tuberculosis* in a BCG prime-boost regimen, female BALB/c mice were boosted with Mpg- or BCG-MNP at week 2 post-BCG vaccination. Another 2 weeks after Mpg- or BCG-MNP boost, the mice were challenged with the virulent *M. tuberculosis* K-strain intravenously, which is a Beijing family member showing hypervirulent features (**Figure 4A**). At week 4 post-challenge, the BCG prime/Mpg-MNP (BCG/MP) group showed a significantly reduced bacterial burden, with low CFUs in the lungs, compared with the BCG prime only or BCG prime/BCG-MNP (BCG/BP) group (**Figure 4B**).

The amelioration of inflammatory cellular accumulation caused by *M. tuberculosis* infection was investigated by histopathological observation. Consistent with the counts of bacteria in the lungs, inflamed pulmonary lesions were decreased in the BCG/MP group compared with all other groups, with alleviation of inflammation and moderate fibrosis (**Figure 4C**). These observations demonstrate that an enhanced protective effect against *M. tuberculosis* infection is conferred by boosting with Mpg-MNP compared to BCG-MNP.

### Induction of Th1-biased immune responses by an Mpg-MNP boost in a virulent *M. tuberculosis* infection model

**Figure 5:**
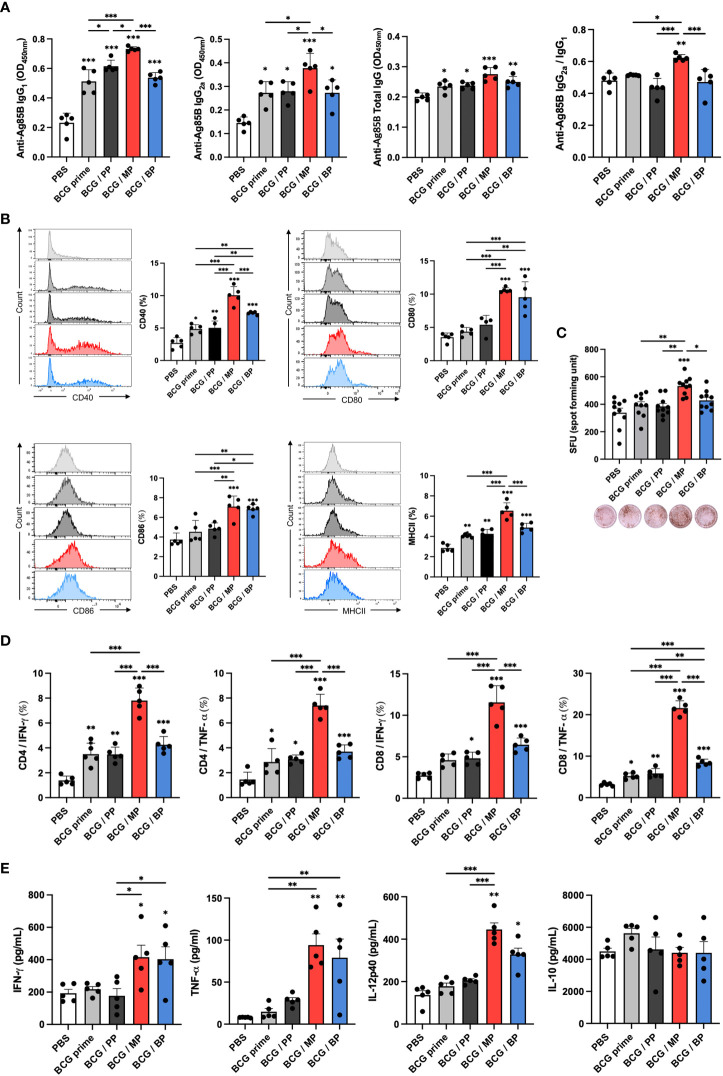
Induction of immune responses against virulent *M. tuberculosis* in splenocytes with a BCG prime/Mpg-MNP boost protocol. **(A)** The levels of IgG_1_, IgG_2a_, and total IgG against the Ag85B protein in the serum were measured by ELISA, and the ratio of IgG2a/IgG1 was determined by dividing the OD values of IgG2a by those of IgG1. **(B)** The graphs show the CD40^+^ (upper left), CD80^+^ (upper right), CD86^+^ (lower left) and MHCII^+^ (lower right) on CD11b^+^ CD11c^+^ dendritic cells among splenocytes. **(C)** The level of IFN-γ -secreting splenocytes restimulated with Ag85B peptides was measured by ELISPOT. The plots of bar graph show two biological replicate for each mouse. **(D)** The results of flow cytometry indicate the populations of IFN-γ - or TNF-α-releasing CD4^+^ (left panel) and CD8^+^ T cells (right panel) among splenocytes that were restimulated with the Ag85B protein for 72 h. **(E)** The bar graphs show the cytokine expression levels in the supernatant of splenocytes restimulated with the Ag85B protein for 72 h, as measured by ELISA. The results show the levels of IFN-γ, TNF-α, IL-12p40, and IL-10 (BCG prime; BCG prime only, BCG/PP; BCG prime/PBS-MNP boost, BCG/MP; BCG prime/Mpg-MNP boost, BCG/BP; BCG prime/BCG-MNP boost). Statistical analysis: One-way ANOVA with Tukey’s multiple comparison test, ^*^P < 0.05, ^**^P < 0.01 and ^***^P < 0.001.

To explain the protective effect of an Mpg-MNP boost after BCG priming against *M. tuberculosis*, the induction of a systemic immune response was analyzed. We evaluated Ag85B-specific IgG subtypes, IgG1 and IgG2a, which are markers of Th2 and Th1 immune responses, respectively, in the sera. Although the BCG/MP group showed levels of Ag85B-specific total IgG similar to those of the other groups, increased levels of both IgG1 and IgG2a were identified in Mpg-MNP-boosted mice, which implies that Mpg-MNP can elicit both Th1 and Th2 immune responses (**Figure 5A**). In particular, the observation that the highest Ag85B-specific IgG2a/IgG1 level was in the BCG/MP group demonstrates that a Th1-biased immune response plays a key role in protection against tuberculosis after BCG vaccination.

Following the induction of the systemic immune response by the Mpg-MNP boost, we further analyzed the altered immune milieu in splenocytes. Increased expression levels of CD40, CD80, CD86 and MHCII were found in the Mpg-MNP boost group compared with the BCG prime only group, suggesting DC maturation (**Figure 5B**). Next, we identified the frequency of IFN-γ-secreting cells among splenocytes; IFN-γ is mainly produced by Th1 cells. Increased IFN-γ spot-forming cells (SFCs) were detected in the BCG/MP group after restimulation with Ag85B protein versus either BCG prime only or BCG/BP group (**Figure 5C**). In addition, as shown by FACS analysis, compared with the BCG prime or BCG/BP group, the BCG/MP group noticeably induced the generation of Ag85B-specific CD4+ and CD8+ T cells expressing IFN-γ, consistent with ELISPOT analysis. Additionally, the frequency of TNF-α-expressing CD4+ or CD8+ T cells was also greatly increased in response to the Mpg-MNP boost (**Figure 5D** and **Supplementary Figure 4**). Therefore, highly activated T lymphocytes expressing both IFN-γ and TNF-α correlated with the strengthening of Th1 immune responses by the Mpg-MNP boost (29). The levels of Th1 immune response-related cytokines, such as IFN-γ, TNF-α, and IL-12p40, secreted by splenocytes, are also indicators of the cell-mediated immune response and were increased in the splenocytes from the BCG/MP group on day 3 after Ag85B restimulation, while similar levels of IL-10 were detected in all groups (**Figure 5E**). These trends were also observed when splenocytes were restimulated with H37Ra lysates, and the abundance of H37Ra-specific IgG subsets and the activation of T cells were increased (**Supplementary Figure 3**). Collectively, these results demonstrate that the BCG prime-boost strategy with Mpg-MNP elicits an improved Th1-biased immune response in a virulent *M. tuberculosis* infection model, suggesting that Mpg-MNP provides enhanced vaccine efficacy as a BCG vaccine booster.

## Discussion

BCG is the only TB vaccine administered worldwide that can reduce the incidence of childhood TB (30). Although BCG vaccination is highly recommended in most countries, TB remains a fatal infectious disease (31). Since BCG vaccination coverage was 85% in 2020 globally (32), the development of a new booster vaccine is mandatory to properly control TB in adults who have previously received the BCG vaccine. It has been proven that the sequential administration of vaccines using different antigens, the so-called heterologous prime-boost vaccine strategy, could be more effective for T-cell-based vaccines against *M. tuberculosis* than the repeated homologous prime-boost vaccine strategy (33). Indeed, *M. indicus pranii* has been shown to provide enhanced protection against *M. tuberculosis* as a BCG booster vaccine by increasing the frequency of multifunctional T cells (34). Based on this concept, we boosted the efficacy of BCG vaccination with Mpg-MNP to control tuberculosis infection by eliciting more enhanced T-cell immunity than BCG-only or BCG-MNP boost.

Microneedles have attracted attention as a promising transdermal immunization strategy (35). Several microneedle systems have been approved by the FDA or been investigated in clinical trials (36). However, most existing dissolving-type microneedles have been manufactured *via* a step-by-step micro-molding method using a three-dimensional (3D) mold and with the use of heat or ultraviolet (UV) light in the curing step (37). It is difficult to fill the mold cavity without drug loss, and such curing steps involve harsh conditions for biological drugs such as vaccines. In the DEN method, by dissolving the microneedle in each droplet, the drug can be loaded into the microneedle without loss of the drug, and the dose of the drug can be easily controlled by controlling the volume and concentration of the drug in the droplet. In addition, since the DEN method does not require molds, it is suitable for mass production and can achieve manufacture within 10 minutes. Following these perspectives, the DEN method provides additional advantages in terms of fabrication costs and the retention of drug biological activity. Consistent with the benefits of the DEN method, the fabrication of Mpg-MNPs showed superior market competitiveness.

Our previous study showed that Mpg is a temperature-sensitive vaccine candidate against tuberculosis, which proved to be safer and more effective in protecting against *M. tuberculosis* and *M. abscessus* in mouse model than BCG (12, 38). Mpg induces a shift in cytokine patterns and antibody production toward a Th1 phenotype and enhances the cytotoxic T-cell response by enhancing DC maturation. In addition, the feasibility of using Mpg as a vaccine vehicle expressing foreign antigens has also been proven in two independent studies (13, 14). Furthermore, live vaccines have shown powerful immunogenicity with induction of long-term immunity by inducing both humoral and cell-mediated immunity based on strong innate immune responses to vital pathogen-associated molecular patterns (vita-PAMPs) (39). Several recent studies have illustrated that cyclic-di-adenosine monophosphate (c-di-AMP) or cyclic-di-guanosine monophosphate (c-di-GMP) produced by live gram-positive bacteria, including *Listeria* spp. and *Mycobacterium* spp., can induce DC activation *via* activation of the innate sensor stimulator of interferon genes (STING)-type 1 interferon (IFN-I) axis, highlighting the importance of maintaining microbial viability in live vaccine formulations (40, 41).

In this study, the high viability of Mpg on the MNPs indicates that Mpg is a more suitable strain for MNPs than BCG. To increase the viability of BCG during microneedle manufacturing, preservatives such as D-(+)-trehalose dihydrate should be added or BCG should be powdered for loading (22, 42). However, such preservatives could cause gastrointestinal discomfort and should be used with caution, especially in insulin users. Additionally, to prepare BCG powder, BCG should be lyophilized at -40°C, which requires more time, leading to reduced manufacturing efficiency. Based on the strong viability of Mpg, we aimed for a process without any preservatives or lyophilization process through the DEN method. Furthermore, Mpg has a faster optimal growth rate than BCG at 30°C, which provides great advantages for the mass production of Mpg-MNPs at low cost, illustrating the benefits of manufacturing. Considering these observations, the superiority of Mpg as a live vaccine makes it a suitable strain for MNP fabrication for tuberculosis protection.

**Figure 6:**
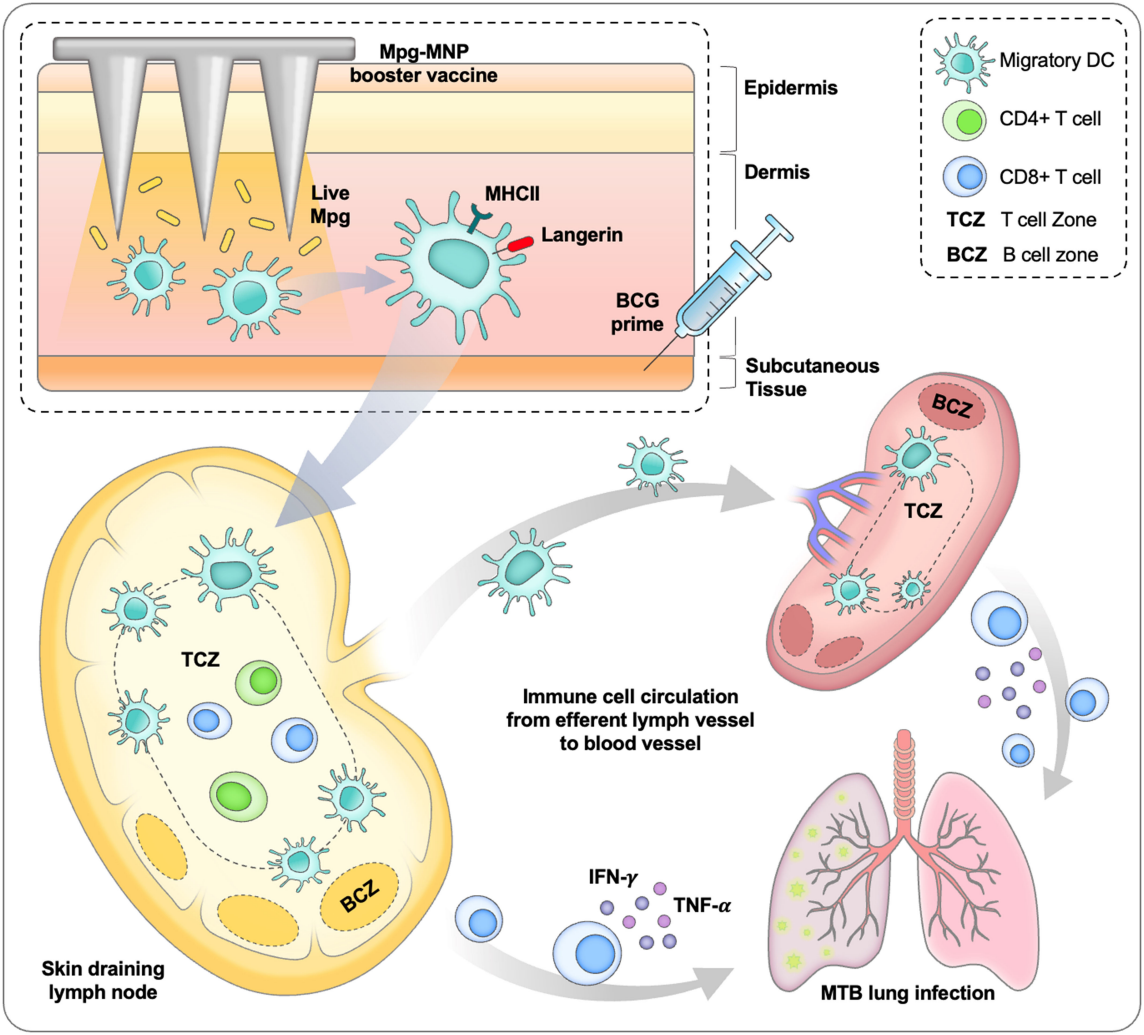
Schematic diagram about protective immune response induced by BCG prime/Mpg-MNP boost strategy.

The microneedle system mainly targets antigen-presenting cells in the skin, which can lead to systemic immunity by activating cellular immune responses in the skin-draining lymph nodes (43). In this study, we identified a large subset of MHCII+ langerin+ cells in the dermis, which are considered a subtype of not Langerhans cells but dermal langerin+ DCs (44–46). Langerin+ dermal DCs express langerin, a C-type lectin involved in the capture of antigens and antigen presentation to T cells, and play an essential role in cell-mediated and Th1 immune responses by migrating to the draining lymph nodes (47, 48). In addition, we detected a robust increase of Ki-67+ langerin+ cells, indicating that the inflammatory environment can induce local regeneration of DCs by renewal, enhancing their response to antigenic challenge and increasing migration to the draining lymph nodes (49, 50).Since the protective effect against *M. tuberculosis* relies on cell-mediated immunity, effector T cells play a pivotal role during *M. tuberculosis* infection, and it is important to boost the efficacy of BCG vaccination in protecting against tuberculosis by activating antigen-specific CD4+ and CD8+ T cells *via* migratory dermal DCs (51). Furthermore, langerin+ dermal DCs are known to play a pivotal role in lung immunity by inducing CD8+ T-cell-mediated immune responses (52). During influenza virus infection, although they have a low frequency among total dermal DCs, depletion of dermal langerin+ DCs impedes pathogen clearance and worsens lung infection (53). Therefore, compared to a single dose of BCG vaccination or BCG-MNP boost, a booster with Mpg-MNP vaccination after BCG priming can enhance the protective effect against *M. tuberculosis* by enhancing cell-mediated immunity *via* activation of Th1 immune responses by proliferating dermal langerin+ DCs. The successful transdermal delivery of live Mpg engages systemic immune responses against *M. tuberculosis* infection *via* activation of antigen-presenting cells, specifically MHCII+ langerin+ cells, migrating into the skin draining lymph nodes. Of note, the induction of cellular immune responses by Mpg-MNP boost has been shown to reduce the pulmonary bacterial burden and ameliorate lung pathology caused by infection with a virulent *M. tuberculosis* strain compared to those observed after BCG priming (**Figure 6**).

In this study, we proposed to introduce Mpg-MNP to overcome the limitations of hypodermic injection through comparison with BCG-MNP and we demonstrated the superiority of Mpg-MNPs to induce local dermal immune system as an effective topical transdermal delivery strategy. On the other hand, due to the lack of delivery efficiency of MNP (**Supplementary Figure 5A**), MNP shows limitation of immune response induction compared to hypodermic injection (**Supplementary Figure 5B**). Since this is the first attempt to apply live mycobacterial-loaded MNP based on the DEN method, further technical optimizing processes will be necessary for MNPs to increase the delivery efficiency of the live Mpg.

In conclusion, our data showed that Mpg-MNP led to enhanced humoral and cell-mediated immune responses against tuberculosis antigens in the BCG prime-boost vaccination strategy, mainly due to enhanced abundance of MHCII+ langerin+ subsets in the dermis of a mouse model. Furthermore, booster vaccination with Mpg-MNP after BCG vaccination led to enhanced protection against infection with the hypervirulent *M. tuberculosis* K-strain in mice, suggesting the feasibility of the HA-based dissolving Mpg-MNP introduced in our study as a candidate for a booster tuberculosis vaccine.

## Data availability statement

The original contributions presented in the study are included in the article/**Supplementary Material**. Further inquiries can be directed to the corresponding authors.

## Ethics statement

The animal biosafety level-2 (BSL-2) experiments were approved by the Institutional Animal Care and Use Committee (IACUC; Approval No. SNU-210531-5) of the Institute of Laboratory Animal Resources of Seoul National University. The BSL-3 experiments were approved by the Institutional Animal Care and Use Committee of Seoul National University Hospital (SNUH-IACUC; Approval No. SNUH IACUC No. 19-0162-C1A1), and animals were maintained in a facility accredited by AAALAC International (#001169) in accordance with the Guide for the Care and Use of Laboratory Animals 8th edition, NRC (2010).

## Author contributions

B-JK and M-HL designed the study and wrote the manuscript. M-HL designed *in vivo* experiments. M-HL and HS performed experiments and analyzed data. BK conceived the conceptualization. M-SL, JS and JJ provided MNPs. HK, DL and JO participated data analysis. B-JK and DJ supervised the study. The authors confirmed and approved the final manuscript. All authors contributed to the article and approved the submitted version.
