# Supplementary material for: Protection against tuberculosis achieved by dissolving microneedle patches loaded with live Mycobacterium paragordonae in a BCG prime-boost strategy
Source: Front Immunol. 2023 Jun 16;14:1178688. doi: 10.3389/fimmu.2023.1178688 (PMC10312308; doi:10.3389/fimmu.2023.1178688)
Supplement: Supplementary file 1 [file DataSheet_1.docx]

Protection against tuberculosis achieved by dissolving microneedle patches loaded with live *Mycobacterium paragordonae* in a BCG prime-boost strategy

**Mi-Hyun Lee ^1,2,6^†, Hyejun Seo ^1,4^†, Moon-Su Lee^7^, Byoung Jun Kim ^1^, Hye Lin Kim^1,4^, Du Hyung Lee^1,4^, Jae Hun Oh^1,2,6^, JuYeop Shin^7^, JuYoung Jin^7^, Dohyeon Jeong^7^* and Bum-Joon Kim^1,2,3,4,5,6^***

*** Correspondence:
*** Dohyeon Jeong, PhD, Raphas Co., Ltd. 62, Magokjungang 8-ro 1-gil, Gangseo-gu, Seoul, Republic of Korea. 07793. [tony@raphas.com](mailto:tony@raphas.com), Telephone: +82-2-3923011, Fax:+82-2-3923012

***** Bum-Joon Kim, PhD, Professor, Department of Biomedical Sciences, Microbiology and Immunology, Liver Research Institute, Cancer Research Institute and SNUMRC, College of Medicine, Seoul National University, 103 Daehak-ro, Jongno-gu, Seoul 03080, Republic of Korea, kbumjoon@snu.ac.kr, Telephone: +82-2-7408315, Fax: +82-2-7430881

##

**Supplementary Figure 1.** **The number and the viability test of mycobacteria loaded-MNPs using qPCR and ATP bioluminescence assay**

**(A)** The standard curve was depicted between the log CFU of Mpg or BCG versus the Cq value of *hsp65*. **(B)** The viable Mpg or BCG on the MNP was measured via the ATP bioluminescence assay. Statistical analysis: One-way ANOVA with Tukey’s multiple comparison tests, ^***^P < 0.001.

**
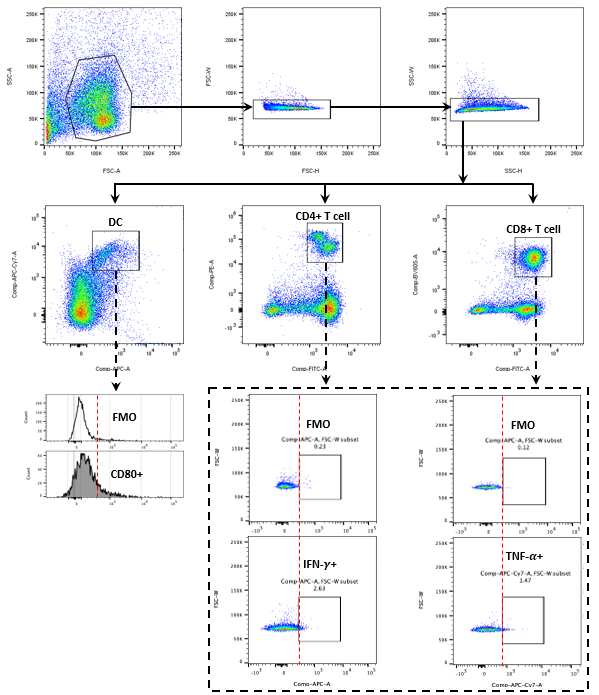
**

**Supplementary Figure 2. The gating strategy for DC or cytokines expressing CD4+ or CD8+ T cells.**

The flow cytometry data of *in vivo* samples was analyzed using FMO (Fluorescence minus one) controls.


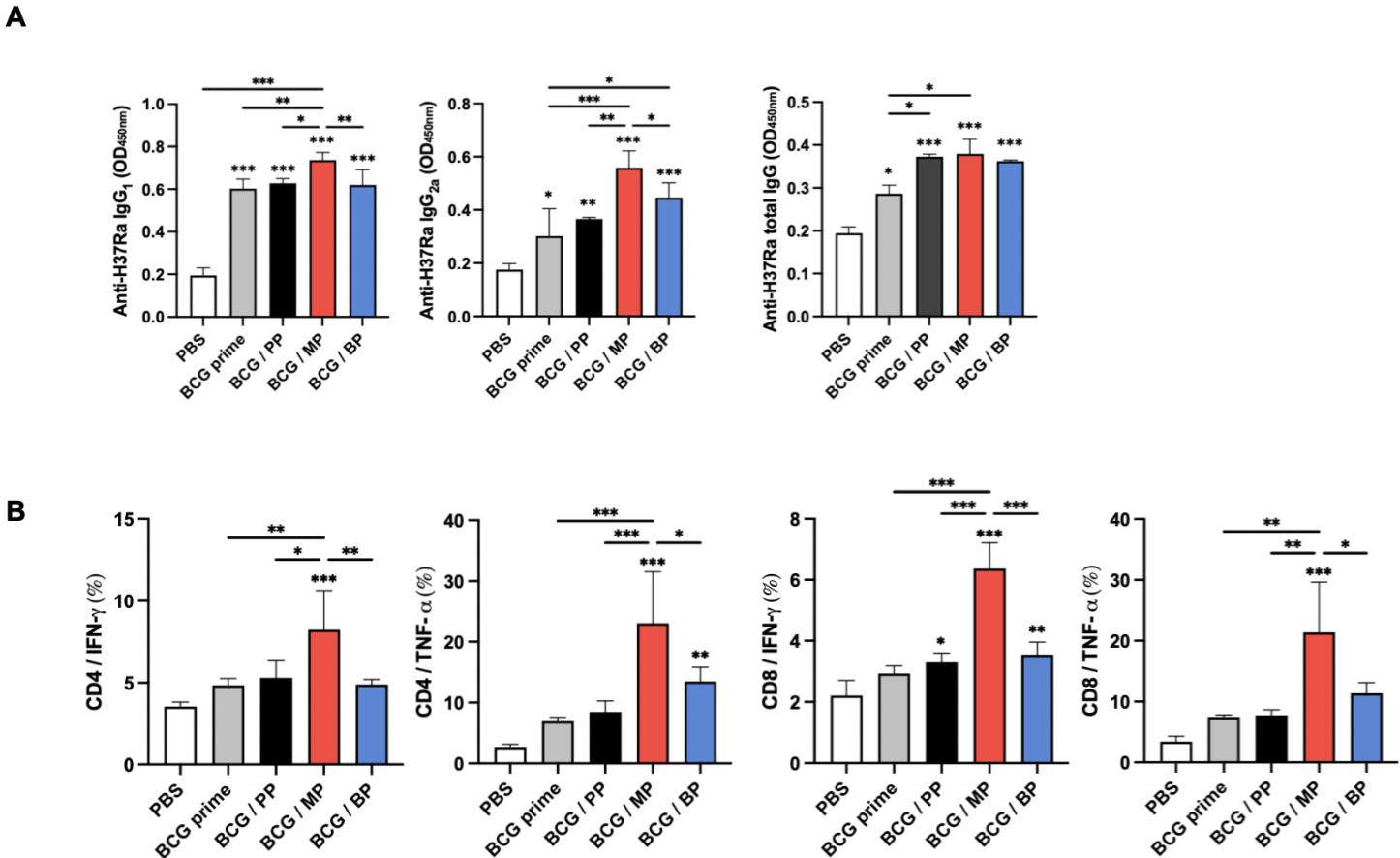


**Supplementary Figure 3. The induction of both humoral and cellular immune responses by an Mpg-MNP boost in a virulent *M. tuberculosis* infection model**

**(A)** The levels of IgG_1_, IgG_2a_, and total IgG against the H37Ra lysate in the serum were measured by ELISA. **(B)** The results of flow cytometry indicate the populations of IFN-γ - or TNF-α-releasing CD4^+^ and CD8^+^ T cells among splenocytes that were restimulated with the H37Ra protein for 72 h. (BCG prime; BCG prime only, BCG / PP; BCG prime/PBS-MNP boost, BCG / MP; BCG prime/Mpg-MNP boost, BCG / BP; BCG prime/BCG-MNP boost). Statistical analysis: One-way ANOVA with Tukey’s multiple comparison test, ^*^P < 0.05, ^**^P < 0.01, and ^***^P < 0.001.

**Supplementary Figure 4. The plot data of IFN-γ - or TNF-α-releasing CD4+ and CD8+ T cells among splenocyte activated with BCG prime/Mpg-MNP boost strategy**

The representative plot data demonstrate IFN-γ - or TNF-α releasing CD4+ or CD8+ T cells of Figure 5D.

**Supplementary Figure 5. Transdermal efficiency of Mpg-MNP versus Mpg-SC and immune response of BCG prime/Mpg boosting strategy following injection route**

**(A)** The left graph shows the log CFU of injected Mpg (Pre) and the homogenized lysate of the back skin from the mouse which was derived one day after being attached with Mpg-MNP or injected with Mpg-SC. The CFU percentage of Post compared to Pre is described as transdermal delivery efficiency and depicted on the right graph. **(B)** The levels of immunoglobulin against Ag85B protein in the serum (upper panel) and the cytokine level of splenocyte stimulated with Ag85B protein for 72h (lower panel) were measured by ELISA (upper panel). (BCG prime; BCG prime only, BCG / MP; BCG prime/Mpg-MNP boost, BCG / BP; BCG prime/BCG-MNP boost, BCG / MSC; BCG prime/Mpg-SC boost, BCG / BSC; BCG prime/BCG-SC). Statistical analysis: One-way ANOVA with Tukey’s multiple comparison test, ^*^P < 0.05, ^**^P < 0.01, and ^***^P < 0.001.

**Table S1. The raw data of mechanical fracture force measured by a universal testing machine**
